# Internal validity of a household food security scale is consistent among diverse populations participating in a food supplement program in Colombia

**DOI:** 10.1186/1471-2458-8-175

**Published:** 2008-05-23

**Authors:** Michelle Hackett, Hugo Melgar-Quinonez, Martha C Alvarez Uribe

**Affiliations:** 1 Department of Human Nutrition, The Ohio State University, Columbus, Ohio, USA; 2 School of Nutrition and Dietetics, University of Antioquia, Medellin, Colombia

## Abstract

**Objective:**

We assessed the validity of a locally adapted Colombian Household Food Security Scale (CHFSS) used as a part of the 2006 evaluation of the food supplement component of the Plan for Improving Food and Nutrition in Antioquia, Colombia (MANA – *Plan Departamental de Seguridad Alimentaria y Nutricional de Antioquia*).

**Methods:**

Subjects included low-income families with pre-school age children in MANA that responded affirmatively to at least one CHFSS item (n = 1,319). Rasch Modeling was used to evaluate the psychometric characteristics of the items through measure and INFIT values. Differences in CHFSS performance were assessed by area of residency, socioeconomic status and number of children enrolled in MANA. Unidimensionality of a scale by group was further assessed using Differential Item Functioning (DIF).

**Results:**

Most CHFSS items presented good fitness with most INFIT values within the adequate range of 0.8 to 1.2. Consistency in item measure values between groups was found for all but two items in the comparison by area of residency. Only two adult items exhibited DIF between urban and rural households.

**Conclusion:**

The results indicate that the adapted CHFSS is a valid tool to assess the household food security of participants in food assistance programs like MANA.

## Background

Food security has been defined as access by all people at all times to enough food, acquired by socially acceptable means, for an active and healthy lifestyle [1]. Moderate, low and very food security consists of situations that range from mild concern over obtaining sufficient amounts of food to coping mechanisms in which the quality and quantity of food consumed is dramatically decreased. Due to various social, economic and physical disparities, over 850 million people worldwide are hungry [2]. At the 1996 World Food Summit in Rome, Italy world leaders set a goal to reduce the number of hungry people in half by the year 2015 [3]. In order to meet this goal, governmental and non-profit agencies in many regions of the world have joined forces to develop programs to reduce food insecurity in high risk populations. One example is Plan for Improving Food and Nutrition in Antioquia, Colombia (MANA – *Plan Departamental de Seguridad Alimentaria y Nutricional de Antioquia*), a nutrition intervention begun by the regional government of Antioquia, Colombia in 2002 for low-income households with pre-school aged children [4]. In 2006, the first extensive evaluation of the food supplement component of this program was spearheaded by Colombian researchers to determine the current nutritional status and food security of MANA participants [5].

The measurement of food security is crucial for governmental and development agencies to monitor and evaluate the impact of their programs at the household level [6]. Historically four measures have been used to measure food security, including national levels of dietary energy supply, individual food intake reports, anthropometry and questionnaires measuring experiences of food insecurity [7]. There are weaknesses in the first three approaches that rely on indicators distinct from the conceptualization of household food insecurity and are costly and time-consuming. Questionnaires included in the last approach fill these gaps and accurately capture and quantify the experiences of food security at the household level, while relatively less inexpensive, easy to use and applicable to diverse populations [8].

For nearly 20 years researchers have created and validated methods to measure food security experiences in questionnaire format [9]. One of the first modules developed for the Community Childhood Hunger Identification Project was based on the Massachusetts Nutrition Survey (1983), in which researchers defined hunger as food insufficiency due to lack of resources [10]. Lorenzana translated this instrument into Spanish, modified the format and validated it with poor peri-urban households in Venezuela [11]. In 2003–2004, researchers in Antioquia, Colombia, conducted a validation study using the adapted Lorenzana tool [Colombia Household Food Security Survey-CHFSS; [12]]. This 12-item survey consists of a range of questions about adult, child and household food security experiences (Table 1). The results of Alvarez's work led to the inclusion of the CHFSS in the 2006 MANA evaluation [5]. The novelty of our research expands the application of household food security surveys and demonstrates the tool's suitability for assessing food assistance programs such as MANA.

**Table 1:** Adapted Colombian household food security survey [CHFSS].

**In the last month**...			**Frequency**		
			
	Yes	No	Always	Sometimes	Rarely
Was there no money to buy food? †					
Did an adult eat less than they wanted because there was not enough money to buy food?					
In the household, was the number of normal meals was decreased, for example not eating breakfast, lunch or dinner because there was no money to buy food?					
Did an adult not eat breakfast, lunch or dinner because there was no money to buy food?					
Did any adult eat less in the main meal because there was not enough food for everyone?					
Did an adult complain of hunger because of lack of food in the house?					
Did an adult go to bed hungry because there was not enough money for the food?					
Did you buy less necessary food items for the children because the money did not last?					
Did any child not eat breakfast, lunch, or dinner because there was not enough money for food?					
Did any child eat less in the main meal because there was not enough food for everyone?					
Did any child complain of hunger because of lack of food in the house?					
Did any child go to bed hungry because there was not enough money for the food?					

† Not included in analysis because it was used as a filter.

When quantifying households by food security status using tools similar to CHFSS, some high risk populations experience more frequent and severe situations of food insecurity than other groups [13]. Previous research demonstrates that rural, low income and large households report higher prevalence of low food security [14,15]. Nevertheless; it is critical to evaluate consistency in the questionnaire's psychometric characteristics between high risk population groups and their less vulnerable counterparts [6,16-18]. The research we present is significant because it explores the variations in questionnaire psychometrics dependent on area of residency, socioeconomic status and number of children participating in MANA. This validation study is a necessary step to develop a household food security survey that can be applied ubiquitously to diverse populations and is critical for food assistance programs similar to MANA that need a valid tool to assess the household food security status of their participants.

## Methods

The psychometric properties of the CHFSS were assessed using data collected from a cross-sectional stratified random sample taken from the total population of MANA participants in Antioquia, Colombia. Sample size was calculated by Colombian researchers using Epitat^® ^software to determine a representative sample of the 200,000 MANA participants. They allowed for a maximal regional error of 0.05% with a resulting sample of 2,784 low-income households with pre-school children. The first item was eliminated from analysis because it was used as a filter. Consequently, households that responded negatively to the first item were removed from the analysis, leaving a maximum possible sample size of 1,319 [19]. The ethics committee at The University of Antioquia approved data collection with informed consent collected once the purpose of the study, dispersion of data, participant rights and risks were explained prior to participation. The analysis of the resulting database was approved by the Institutional Review Board at the Ohio State University.

### Rasch model

Researchers in the US have recommended the Rasch Model to develop household food security surveys and evaluate the psychometric characteristics of their items [20]. The Rasch Model belongs to a family of item-response-theory (IRT) statistical scaling models that fits questionnaire items measuring the same underlying construct along a logit continuum [21]. The resulting intervals between items and order alert survey designers to potential problems with the items, their order within the questionnaire and score interpretations from the data [19]. Numerous validation studies of adapted household food security questionnaires including the US Household Food Security Survey Model have been done using Rasch Modeling techniques [12,16-18,22-26].

The Rasch Model assumes that the items within the questionnaire are one-dimensional, measure the same construct, and are independent of one another [19]. The first two assumptions are assessed by FIT statistics, which measure the difference in the expected and the actual responses [27]. These values are estimated by squaring the difference between actual and modeled responses, summing the squared differences of all items, averaging the sum and then standardizing the results to approximate a unit normal (z) distribution [28]. For our study, weighted item INFIT values were assessed which are sensitive to unexpected behavior that affects responses to items near the person's ability level and are less sensitive to extreme responses. When the responses fit the model perfectly, the resulting item INFIT value is 1.0, with a recommended range of 0.8 to 1.2 and a wider acceptable range of 0.7–1.3 [23]. Item INFIT values above one demonstrate that the respondents performed too well on the item in comparison to their total scores. When item INFIT values are below one, fewer individuals responded affirmatively to the item than would be expected based on the order in the questionnaire and suggest item redundancy [23]. In general, item misfit may result from items that are too complex, confusing or measuring a different construct [28].

Assessment of survey item independence is done using a second statistical outcome of Rasch modeling called measure values that demonstrate the relative severity of each of the questions in correspondence to the actual food insecurity status of the interviewees. This outcome is possible because Rasch Model assumes that the higher the severity of the item, the less likely it will be answered affirmatively; and the more food insecure the household, the more likely the respondent will answer affirmatively to each question [17]. Measure values are quantified using the natural log of the odds of the respondent successfully answering the items within the food security questionnaire and are compared along a logit continuum [27]. Measure values allow researchers to evaluate the spread of items along the questionnaire continuum and identify areas of food insecurity that are poorly quantified by the items [29]. Any large gaps along the measurement value continuum indicate that additional items are needed to distinguish within that particular range of severity. If two different items have the same measurement value, this likely means that the items are measuring the same level and indicates that one of the questions might be dropped in order to decrease the respondent load.

When the conditions of the Rasch Model have been met, unidimensionality of a scale can be assessed using Differential Item Functioning [DIF; [30]]. DIF allows comparisons across groups while holding the level of psychological disturbances constant. A DIF contrast greater than 0.5 logit units is considered substantial and demonstrates that response probabilities are not fully explained by the latent trait [31]. This means that other variables are influencing the response and make comparisons between groups problematic. DIF effects are computed in Winsteps (Winsteps, Chicago, IL) by subtracting the measure values for two groups and then converting the differences to standard normal variates using a pooled standard error [32].

To fit the data to the Rasch Model, responses to the items were coded as "yes" = 1 and "no" = 0. The follow-up frequency items were incorporated into the original questions as follows: if the individual responded "yes" to the first question and responded "almost every day" or "on just a few days" to the frequency question, they remained classified as 1. On the other hand, if the respondent answered "yes" to the first answer and "on only one or two days" to the frequency question, they were reclassified as 0 [33].

After initial fitting a Rasch Model was done with all households, the complete databases were separated into reference and secondary groups to compare psychometric characteristics of specific sub-populations within this sample. Reference groups followed by secondary groups are as follows:

• One child participating in MANA (n = 713); Multiple children participating in MANA (n = 604)

• Very low income (n = 789); Low income (n = 481)

• Urban (n = 560); Rural (n = 759)

We were interested in differences in CHFSS performance between households characterized by very low income and low income, localization in urban and rural areas and one versus multiple children participating in MANA. Households were eliminated from the socioeconomic status analysis that reported an income above level for normal admittance into MANA or missing data (n = 35). There were two households not included in the one versus multiple children analysis that had missing child data. Winsteps 3.52 (Winsteps, Chicago, IL) was used to conduct Rasch Model analysis with the XMLE = yes command to correct for estimate bias

## Results

As shown in table 2, item INFIT values were within the appropriate range (0.8 to 1.2) for all but two child items. In that regards, for the low income group the child item *went to bed hungry *was the only item with an INFIT value below 0.8 (INFIT = 0.78), but still within an acceptable range (0.7 – 1.3). In addition, the child item *buy less staples *had an INFIT value within a range of 1.17–1.36, where only the values for the low income and rural subgroups were within the 0.8–1.2 range. This item was also outside the wider acceptable range of 0.7–1.3 for very low income and urban households (INFIT = 1.31 and 1.36, respectively).

**Table 2:** Infit values for adult and child items by groups [n = 1,319].

		Children Participating in MANA		Socioeconomic Status		Area of Residency	
**Adult Items**	All	One Child	Multiple Children	Very low income	Low income	Urban	Rural

Decreased meals	1.09	1.09	1.10	1.10	1.03	1.03	1.14
Ate less	0.93	0.91	0.95	0.87	1.03	0.91	0.95
Skipped meal	0.87	0.91	0.82	0.92	0.84	0.86	0.89
Ate less – main meal	0.94	0.95	0.93	0.91	1.00	0.95	0.94
Hungry	1.03	1.01	1.04	1.01	1.05	1.07	1.00
Went to bed hungry	0.94	0.91	0.98	0.99	0.84	0.91	0.97

**Children Items**							

Buy less staples	1.26	1.27	1.25	1.31††	1.17	1.36††	1.19
Skipped meals	0.88	0.88	0.90	0.89	0.87	0.90	0.86
Ate less – main meal	1.07	1.07	1.08	1.09	1.06	1.03	1.09
Hungry	0.9	0.90	0.92	0.83	0.98	0.84	0.93
Went to bed hungry	0.82	0.81	0.81	0.81	0.78†	0.80	0.84

† Outside of 0.8 to 1.2 range but within acceptable range of 0.7 to 1.3.

†† Outside of acceptable range of 0.7 to 1.3.

**Figure 1:**
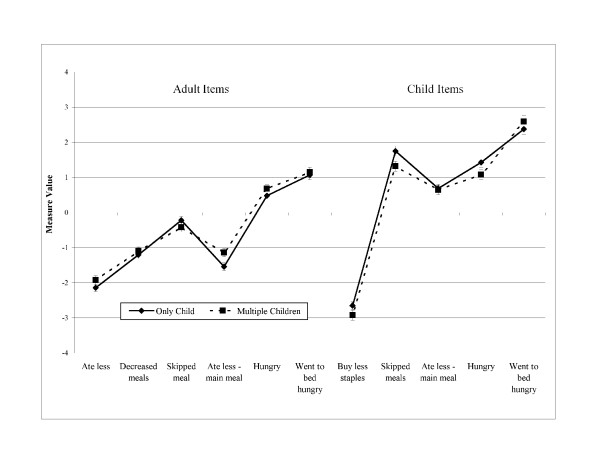
Adapted Colombian household food security survey [CHFSS] item measure values by number of children enrolled in MANA [n = 1,319].

**Figure 2:**
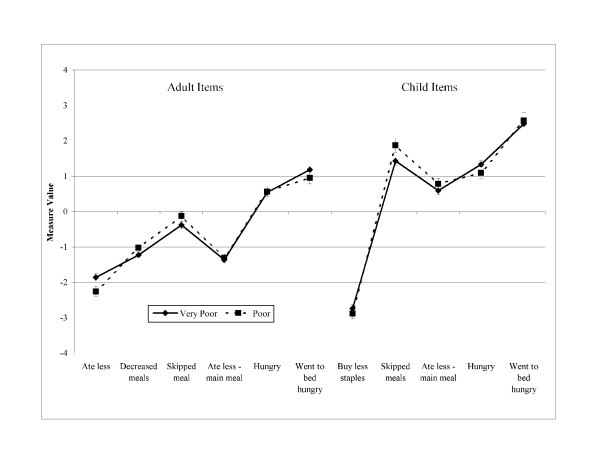
Adapted Colombian household food security survey [CHFSS] item measure values by socioeconomic status [n = 1,319].

**Figure 3:**
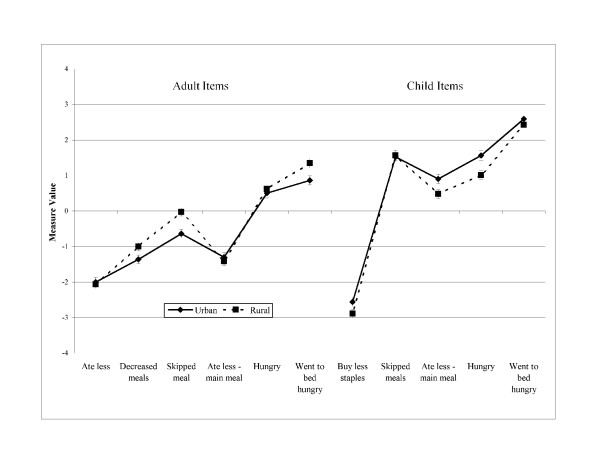
Adapted Colombian household food security survey [CHFSS] item measure values by area of residency [n = 1,319].

Figures 1, 2 and 3 illustrate the order of measure values severity for adult and child stratified by number of children in the household, income status, and area of residency, respectively. Even though the order of measure values is different than the order in which the items are presented in the questionnaire, in all cases a trend is observed where conceptually less severe items had lower measure values than those items representing the more severe underlying conditions. Differences between the order of measure values and the order in which the items are in the questionnaire refer to two items: 1) within the group of adult related items, the item *ate less – main meal (4) *presented a measure value lower than the previous item; and 2) the children item *skipped meals (8) *had a higher measure value than the items *ate less – main meal (9) *and *hungry (10)*. Items were well spread along the measure value continuum for all groups with no gaps.

DIF analysis showed only two items with DIF between urban and rural households for the adult item *skipped meals *(DIF = -0.61; t = -3.64) and the child item *hungry *(DIF = 0.56; t = 2.89). The remaining adult and child items supported CHFSS unidimensionality among the other subgroups.

## Discussion

The item INFIT values demonstrate that the items measure the same construct and are independent of one another with the exception of the child items *buy less staples *and *went to bed hungry*. The low item INFIT *went to bed hungry *suggests redundancy in the less poor socioeconomic group, but the value was still within the wider acceptable range. It would not be advisable to remove these items merely because the item INFIT values were below 0.8 or above 1.2, especially for *skipped meal *where "misfit" was only found in one group. A previous CHFSS validation study in Antioquia, Colombia using a representative sample of 1,624 households had all INFIT values within 0.8 and 1.2 [12].

When the US Core Food Security Measure, a precursor to the USDA Household Food Security Survey Module, applied in Hawaii was analyzed with Rasch, all 15 INFIT values were within the 0.8 to 1.2 range [17]. Adolescent respondents to a 6-item household food security survey in Trinidad and Tobago resulted in INFIT values between 0.798 and 1.132. The only item outside of the strictest range was *cut size or skipped meals *[24]. In Campinas, Brazil, the USDA Household Food Security Survey Module was translated to Portuguese and adapted for cultural acceptability using in-depth focus groups [34]. The resulting 15-item Brazilian Household Food Security Scale (EBIA) was then applied to a regionally representative population in Campinas, Brazil, and analyzed with Rasch, resulting in adult and child items INFIT values within 0.8 and 1.2 for all items except for the adult item *hungry *[25]. The results of this study confirmed the tool's validity and led to its inclusion in the 2004 National Household Sample Survey (*Pesquisa Nacional por Amostra de Domicílios *– PNAD), where it was expanded to 16-items by the Brazilian Institute of Geography and Statistics [18]. The INFIT values we present were better centered within the INFIT range of 0.8–1.2 than female and male respondents in Brazil where three adult *(worried*, *ate less*, and *lost weight] *and two child items *[not enough *and *reduced meal size) *were outside of the range [18].

To our knowledge this is the first large scale DIF analysis to assess cross sample unidimensionality of a household food security survey in a population receiving food assistance. Analysis of food security using the Rasch Model in the US by subgroups of race/ethnicity, household composition, metropolitan status and region of country revealed consistent patterns in item measure values [35]. Previous work in Bangladesh with a locally developed household food security survey had four items with DIF by groups of land ownership status in their scale [36]. As previously shown, DIF was found for the adult item *skipped meals *and the child item *hungry *when comparing urban and rural subgroups in Colombia. The size of the differences between urban and rural households when responding to these items (DIF = 0.61 and 0.56, respectively), can mean that these items are understood differently by the two subgroups, or that the items are tapping into different underlying conditions (36). In addition, differences in the prevalence of positive response to these items could be given by a difference in how severely these items are experienced by each of the subgroups. Adult item *skipped meals *seems to be perceived as more severe (less commonly experienced) for rural households. On the other hand, the child item *hungry *seems to be more severe among urban households, meaning it is experienced less commonly in these areas than in rural settings. Nevertheless, in our opinion the differences found do not suggest that these items should be discarded from the scale. The INFIT and measure values demonstrate good psychometric properties for urban and rural households comparable to the ones found in other subgroups. At this point, we recommend that additional qualitative studies are conducted to assess face validity of these items among urban and rural households. This process might result in a better adaptation of the language and wording of such items, improving its comparability across diverse population groups.

Adult and child item measure value results coincide with the conceptualization of food security as a managed process where minor modifications in dietary intake precede drastic decreases in consumption [37]. Our results show that adults will decrease the amount of food at a given meal prior to reducing or skipping meals. Likewise children will eat less food in the main meal before going hungry and skipping meals. It appears that child buffering occurs within this population where decreases of child food intake only occur after adults decrease their food consumption. This pattern is not consistent with results from the study in Antioquia Colombia where each item followed the pattern of increasing measure value from beginning to end of the questionnaire [12]. These differences are likely the result of changes in item order between the applications of the survey.

The order of measure values did not correspond to question order within the food security questionnaire administered in Hawaii [17]. Specifically, five items were out of questionnaire order based on measure values. Brazilian measure values followed an appropriate increasing pattern for adult and child items when analyzed separately, with the exception of one adult item, suggesting that the order of items they used may correspond better with the conceptualization of each item representing a more severe situation of food insecurity within the questionnaire [25]. The variation between the order of the survey items and the actual measure value of each item suggests the need for a change in item order for the CHFSS to match the underlying construct, so that the items follow the specific order of severity. Additional research is needed to determine the implications of modifying item order within the questionnaire when considering questionnaire item flow.

Rasch Modeling revealed no gaps in CHFSS item measure values. Previous research in Colombia revealed a gap between the first two items and remaining items. These results suggest that the CHFSS better differentiates between food security items with our population than a representative sample from the same region of Colombia [12]. The Colombian adult and child measure values performed better than all US items used with a Hawaiian population, which had three gaps and the US national results of 4 gaps in survey measure values [17]. Researchers used a short household food security form in Trinidad & Tobago with 286 households in which Rasch measure values showed a generally increasing value as the severity of the question increased as the Colombian items did [38]. Good spread of measure values was found at the low end of the short survey, but there were two gaps in measure values where households were not well distinguished between the items. Using a short household food security survey in Trinidad & Tobago with 1,903 students, Rasch measure values showed a generally increasing value as the severity of the questions increased, as the Colombian items did [24]. There was one gap between items *food didn't last *and *cut/skipped meals *between boys and girls across the three ethnic groups of Afro-Caribbean, Indo-Caribbean and Mixed. The Brazilian and US tools revealed that all items had similar measure values with similar trends of increasing severity for both tools with less gaps in the Brazilian analysis than in the Colombian [25]. The Brazilian EBIA child item measure values revealed two clusters of two items: *decreased quality *with *not enough *and *skipped meal *with *hungry *[18]. Similarly, we had two clusters but with adult items towards the more severe end of the measure value spectrum.

## Conclusion

Our results indicate that the adjusted version of the household food security scale is valid for application to diverse low-income households in Colombia, especially in describing the situation of households experiencing severe food insecurity. Additional work is needed to compare the psychometric properties of the tool when applied to program participants versus non-participants. As the CHFSS continues to be validated with new populations, improvements in the tool can be made to capture the actual experience of food insecurity at the household level of MANA program participants. Based on our results, this tool can be used in future program evaluations, thus the CHFSS can play a critical part in policy planning in Colombia. Although this is the first time the CHFSS was used to assess a food supplement program in Colombia, our findings suggest its' suitability for other food assistance programs.

## Competing interests

The authors declare that they have no competing interests.

## Authors' contributions

MH performed the statistical analysis and wrote the manuscript. HM–Q provided advice for statistical analysis and explained the theoretical framework and reviewed all manuscript drafts. MCAU coordinated the data collection organization and controlled the quality of the database. All authors read and approved the final manuscript.

## Pre-publication history

The pre-publication history for this paper can be accessed here:


